# 2124: Three stages of cultural change in translational science

**DOI:** 10.1017/cts.2017.159

**Published:** 2018-05-10

**Authors:** Joseph A. Kotarba

## Abstract

OBJECTIVES/SPECIFIC AIMS: This report describes the evolution of scientific culture since the NIH/translational science (TS) mandate. The transition of the conduct of science to an increasingly translational model involves 2 dimensions of change. The first dimension consists of change in the structure and process of scientific work, in terms of factors such as funding, administration, application of new knowledge, and so forth. The second dimension consists of change in culture of scientific work. The culture of science is the set of values, assumptions, meanings, and traditions that inform the conduct of science. As part of the comprehensive evaluation of TS at the University of Texas Medical Branch-Galveston, we have monitored the status of the culture of science there through a sociological framework. We focused on the ways the changing culture of science facilitates and/or inhibits creative and effective medical research. We argue that the long-term success of TS is dependent upon the evolution of assumptions, everyday practices, and taken-for-granted ways of conducting research. Culture also provides meanings for who its people are and helps us define who we are to ourselves (ie, self-concept). In terms of the scientific enterprise, self-identity provides the motivation to participate in group activities or to be content with being a “lone ranger” researcher; the orientation to be either a leader or a follower; the security to take creative chances with one’s work or to simply conduct “normal science”; and the sense of esteem for being the best or simply doing one’s job. TS requires a constant “reengineering” of its total enterprise. Consequently, we raised the following research questions: (1) What is the traditional culture of science at UTMB? (2) How has the culture of science at UTMB changed since the introduction of the Clinical and Translational Science Award project? (3) What has been the relationship between the culture of science and the conduct of science at UTMB since CTSA? (4) How have cultural influences on self-concept changed? METHODS/STUDY POPULATION: Data have been collected by means of ongoing 1-on-1 interviews with CTSA participants at all levels; observations of lab and classroom interaction; participation in organizational and planning committees; and other everyday organizational activities. RESULTS/ANTICIPATED RESULTS: Following the grounded theory method of qualitative analysis and discovery, we found 3 stages of cultural change. Stage 1 is Cultural Invasion of the existing culture at UTMB by the implementation of the CTSA project. Stage 2 is Cultural Accommodation by which internal responses to change follow the normal scientific paradigm. Stage 3 is Cultural Expansion by which the organizational and cultural platform for conducting science has expanded regionally, nationally and cross-disciplinarily. DISCUSSION/SIGNIFICANCE OF IMPACT: Whether a distinct fourth stage emerges depends on such factors as funding and programmatic directives from NIH; the tension between research and clinical demands for resources; and the emergence of junior investigators schooled on the principles of TS.

